# Identification of an m6A-Related lncRNA Signature for Predicting the Prognosis in Patients With Kidney Renal Clear Cell Carcinoma

**DOI:** 10.3389/fonc.2021.663263

**Published:** 2021-05-26

**Authors:** JunJie Yu, WeiPu Mao, Si Sun, Qiang Hu, Can Wang, ZhiPeng Xu, RuiJi Liu, SaiSai Chen, Bin Xu, Ming Chen

**Affiliations:** ^1^ Medical College, Southeast University, Nanjing, China; ^2^ Department of Urology, Affiliated Zhongda Hospital of Southeast University, Nanjing, China; ^3^ Department of Urology, Affiliated Lishui People’s Hospital of Southeast University, Nanjing, China

**Keywords:** prognostic signature, The Cancer Genome Atlas, long non-coding RNA, kidney renal clear cell carcinoma, M6A

## Abstract

**Purpose:**

This study aimed to construct an m6A-related long non-coding RNAs (lncRNAs) signature to accurately predict the prognosis of kidney clear cell carcinoma (KIRC) patients using data obtained from The Cancer Genome Atlas (TCGA) database.

**Methods:**

The KIRC patient data were downloaded from TCGA database and m6A-related genes were obtained from published articles. Pearson correlation analysis was implemented to identify m6A-related lncRNAs. Univariate, Lasso, and multivariate Cox regression analyses were used to identifying prognostic risk-associated lncRNAs. Five lncRNAs were identified and used to construct a prognostic signature in training set. Kaplan–Meier curves and receiver operating characteristic (ROC) curves were applied to evaluate reliability and sensitivity of the signature in testing set and overall set, respectively. A prognostic nomogram was established to predict the probable 1-, 3-, and 5-year overall survival of KIRC patients quantitatively. GSEA was performed to explore the potential biological processes and cellular pathways. Besides, the lncRNA/miRNA/mRNA ceRNA network and PPI network were constructed based on weighted gene co-expression network analysis (WGCNA). Functional Enrichment Analysis was used to identify the biological functions of m6A-related lncRNAs.

**Results:**

We constructed and verified an m6A-related lncRNAs prognostic signature of KIRC patients in TCGA database. We confirmed that the survival rates of KIRC patients with high-risk subgroup were significantly poorer than those with low-risk subgroup in the training set and testing set. ROC curves indicated that the prognostic signature had a reliable predictive capability in the training set (AUC = 0.802) and testing set (AUC = 0.725), respectively. Also, we established a prognostic nomogram with a high C-index and accomplished good prediction accuracy. The lncRNA/miRNA/mRNA ceRNA network and PPI network, as well as functional enrichment analysis provided us with new ways to search for potential biological functions.

**Conclusions:**

We constructed an m6A-related lncRNAs prognostic signature which could accurately predict the prognosis of KIRC patients.

## Introduction

Renal cell carcinoma (RCC) was the third most common malignant tumor of the urinary system worldwide (1), of which kidney renal clear cell carcinoma (KIRC) was the most frequent subtype (2). Despite the development of many targeted drugs and immunosuppressive drugs, radical nephrectomy was still the primary and most effective treatment method (3). Moreover, KIRC was insensitive to chemotherapy and radiotherapy and had a higher rate of recurrence and metastasis than other subtypes of RCC (3, 4). A better understanding of the molecular mechanisms of KIRC was crucial for the development of new therapeutic agents. It was urgent to identify an effective prognostic signature to predict the survival outcomes of KIRC patients.

DNA methylation and post-translational histone modifications were involved in the epigenetic regulation of cell development and differentiation (5). N6-methyladenosine (m6A) modification was the most abundant internal epistatic modification of mRNA and non-coding RNA (6) and was involved in many biological processes, including RNA splicing, export, and translation (7). The m6A modifications were regulated by m6A regulators, including methyltransferases complex (“writers”), signal transducers (“readers”), and demethylases (“erasers”) (8). It has been reported that M6A was closely associated with a variety of tumors and was thought to be one of the drivers of tumorigenesis and progression. Cai et al. (9) reported that m6A Methyltransferase METTL3 promoted the growth of prostate cancer by regulating hedgehog pathway. Guo et al. (10) reported that RNA demethylases ALKBH5 prevented pancreatic cancer progression by post-transcriptional activation of PER1. Furthermore, m6A-regulated genes also played an essential role in the pathogenicity of KIRC. Zhuang et al. (11) reported that FTO suppressed KIRC progression through the FTO-PGC-1α signaling pathway. Gao et al. (12) reported that DMDRMR-mediated regulation of CDK4 promoted KIRC progression through m6A reader IGF2BP3.

Long non-coding RNAs (lncRNAs) were a class of RNAs that could not encode proteins and have been widely studied in recent years (13). lncRNAs were involved in various biological processes in eukaryotes, and their aberrant expressions were near related to tumor malignancy, including tumor proliferation, differentiation, apoptosis, drug resistance, and metastasis (14, 15). Nevertheless, whether m6A modification-related lncRNAs could be involved in the progression of KIRC remained to be elucidated. Therefore, it was urgent to identify m6A-associated lncRNAs biomarkers for the early diagnosis and prognosis of patients with KIRC.

Here, based on the data of KIRC patients downloaded from The Cancer Genome Atlas (TCGA) database, we constructed an m6A-related lncRNAs prognostic signature by bioinformatic and statistical analysis to predict the prognostic outcomes of KIRC patients accurately. We found that the prognostic signature constructed with five m6A-associated lncRNAs had a high predictive ability. Moreover, a nomogram was constructed to predict the overall survival (OS) of KIRC patients quantitatively. Finally, a ceRNA network and PPI network were built to further explore the possible biological mechanisms of lncRNAs in preparation for identifying new biomarkers.

## Methods

### Data Source and Preparation

As the flow chart of the study shown in **Figure S1**, we downloaded Transcriptome profiling data in fragment per kilobase method (FPKM) format of 530 KIRC patients from TCGA data portal (https://portal.gdc.cancer.gov/). Subsequently, these data were collated and annotated, and then collapsed into protein-coding genes and long non-coding RNAs employing the Ensembl human genome browser (http://asia.ensembl.org/info/data/index.html) using the Perl program (16). And 14,142 lncRNAs were identified. Then, the differential analysis of these lncRNAs was performed by the “limma” package in R 4.0.3 (logFC > 1 or<-1, p < 0.05), and 4,492 significantly differential lncRNAs were identified. In addition, 35 m6A-related genes were obtained from published articles (8, 17), and the expression matrixes were extracted from transcriptome profiling datasets, including regulators on writers [KIAA1429 (VIRMA), METTL3, METTL14, WTAP, RBM15, RBM15B, METTL16, ZC3H13, and PCIF1], readers [TRMT112, ZCCHC4, NUDT21 (CPSF5), CPSF6, CBLL1 (HAKAI), SETD2, HNRNPC, HNRNPG (RBMX), HNRNPA2B1, IGF2BP1, IGF2BP2, IGF2BP3, YTHDC1, YTHDF1, YTHDF2, YTHDF3, YTHDC2, SRSF3, SRSF10, XRN1, FMR1 (FMRP), NXF1, and PRRC2A], and erasers (FTO, ALKBH5, and ALKBH3). The differential analysis was also performed by the “limma” package in R software and 25 m6A-related genes were confirmed to be significantly different (p < 0.05, **Figure S2**). Then, Pearson correlation analysis between these lncRNAs and 25 m6A-related genes was performed, and 753 m6A-related lncRNAs were identified (cor > 0.5 or <−0.5, p < 0.05). The clinicopathological data were downloaded from the TCGA dataset, excluding those with survival time <30 days or unknown (n = 17), and those with unclear specific information including stage (n = 3), tumor grade (n = 3), and AJCC M stage (n = 3). Subsequently, we merged lncRNAs expression data with clinical data. Ultimately, a total of 505 cases were included in the study.

### Construction and Verification of an m6A-Related lncRNAs Prognostic Signature

To construct an effective prognostic prediction signature, we randomly classified the 505 cases into training set (253 samples) and testing set (252 samples) in a 1:1 ratio (**Table 1**). The training set was applied to construct a prognostic signature and to evaluate it in the testing set. The univariate Cox proportional hazards regression analysis was used to identify m6A-related lncRNAs, which were significantly linked with prognosis (p < 0.01) in the training set. Least absolute shrinkage and selection operator (LASSO) regression analysis was applied to eliminate those prognostic-related lncRNAs highly correlated with each other to avoid overfitting. Later, the multivariate Cox proportional hazards regression analysis was subjected to determine independent prognostic factors. Ultimately, we identified five prognostic risk-related lncRNAs to construct a prognostic risk score signature. The risk score of KIRC patients was calculated using the format $risk\;score=\sum_{i=1}^{n}coef(i)*lncRNA(i)\;expression.$ The KIRC patients were classified into high-risk subgroup and low-risk subgroup based on median risk score as the cut-off value. The Kaplan–Meier survival curve was performed to compare the survival outcomes of the two groups. The receiver operating characteristic curves (ROC) and its area under the curve (AUC) values were utilized to evaluate the specificity and sensitivity of the signature by “ROC package” in R software.

**Table 1:** Comparison of clinical characteristics of KIRC* patients in training set and testing set.

Covariates	Type	Overall set	Training set	Testing set	P-value
age	<=60	258 (51.09%)	122 (48.22%)	136 (53.97%)	0.2291
	>60	247 (48.91%)	131 (51.78%)	116 (46.03%)	
gender	FEMALE	173 (34.26%)	90 (35.57%)	83 (32.94%)	0.5958
	MALE	332 (65.74%)	163 (64.43%)	169 (67.06%)	
grade	G1–2	228 (45.15%)	119 (47.04%)	109 (43.25%)	0.6466
	G3–4	272 (53.86%)	132 (52.17%)	140 (55.56%)	
	GX	5 (0.99%)	2 (0.79%)	3 (1.19%)	
stage	Stage I– II	306 (60.59%)	157 (62.06%)	149 (59.13%)	0.5604
	Stage III–IV	199 (39.41%)	96 (37.94%)	103 (40.87%)	
T	T1–2	324 (64.16%)	165 (65.22%)	159 (63.1%)	0.6859
	T3–4	181 (35.84%)	88 (34.78%)	93 (36.9%)	
M	M0	404 (80%)	203 (80.24%)	201 (79.76%)	0.9896
	M1	77 (15.25%)	38 (15.02%)	39 (15.48%)	
	MX	24 (4.75%)	12 (4.74%)	12 (4.76%)	
N	N0	228 (45.15%)	120 (47.43%)	108 (42.86%)	0.0768
	N1	15 (2.97%)	11 (4.35%)	4 (1.59%)	
	NX	262 (51.88%)	122 (48.22%)	140 (55.56%)	

*KIRC, kidney renal clear cell carcinoma.

### Establishment and Validation of a Prognostic Nomogram

To quantitatively predict the prognosis of KIRC patients, we constructed a prognostic nomogram based on risk score and traditional prognosis-related clinical variables, including age, grade, AJCC T stage. Afterward, the concordance index (C-index) and calibration curves were used to evaluate the reliability and accuracy of the prognostic nomogram.

### Gene Set Enrichment Analysis (GSEA) and Weighted Gene Co‐Expression Network Analysis (WGCNA)

GSEA software was performed to explore the potential biological processes and cellular pathways in the low- and high-risk subgroups in KIRC TCGA cohort. The expression profiles of mRNAs and lncRNAs of KIRC patients downloaded from the TCGA database were applied to construct gene co-expression networks using the “WGCNA package” implemented in R software. The construction process was the same as described previously (18). The FPKM method was used to standardize the data. The parameter settings of the dynamic tree cut method referred to previous literature.

### CeRNA Network Construction and PPI Analysis, As Well As Functional Enrichment Analysis

Previous literature has reported potential interactions between mRNAs, miRNAs, and lncRNAs, and to elucidate the regulatory role of m6A-related lncRNAs, we constructed a ceRNA network based on WGCNA and differentially expressed lncRNAs. The lncRNA and mRNAs modules with the highest correlation coefficient were selected. To further close the relationship with the clinical traits and increase the accuracy of prediction, the lncRNAs in the MEturquoise module were intersected with the differentially expressed lncRNAs in the KIRC dataset in the TCGA database, and 12 lncRNAs were finally selected as m6A-associated lncRNAs. The miRcode (http://www.mirco de.org/) database was utilized to predict miRNAs that interacted with 12 lncRNAs, identifying 161 pairs of interactions between 12 lncRNAs and 35 miRNAs. The relationship between miRNAs and target mRNAs was predicted by TargetScan (http://www.targe tscan.org/), miRDB (http://www.mirdb.org/miRDB/), and miRTarBase (http://mirtarbase.mbc.nctu.edu.tw), and 149 mRNAs were identified. Cytoscape software was used to visualize the lncRNA/miRNA/mRNA ceRNA network. STRING (https://string-db.org/) was a website that could predict interactions between functional proteins (19, 20). Those 149 target mRNAs were applied to establish a PPI network. A medium confidence of >0.4 was considered significant. CytoHubba plugin of Cytoscape was used to extract hub genes from the PPI network. Subsequently, using the “clusterProfiler package” in R software, Gene Ontology (GO) enrichment analysis of the 149 targeted mRNA was used to identify molecular functions (MF), cellular components (CC), and biological processes (BP). The Kyoto Encyclopedia of Genes and Genomes (KEGG) was performed to search for potential signaling pathways.

### Cell Lines, Clinical Samples Collection, RNA Extraction, and Quantitative Real-Time Polymerase Chain Reaction (qRT-PCR)

The human KIRC cell lines,786-O, caki-1, and human kidney cell (HK-2 cell, proximal tubule epithelial cell) were originally purchased from cell repository of Shanghai Institute of Life Sciences, Chinese Academy of Sciences. RPMI 1640 medium, containing 10% fetal bovine serum (FBS), penicillin (25 U/ml), and streptomycin (25 mg/ml), was used to culture these KIRC cells at 37°C in a humidified 5% CO2 environment. In addition, a total of 25 fresh samples from patients who underwent laparoscopic radical or partial nephrectomy for KIRC were collected in Southeast University Zhongda Hospital from 2019 to 2020, including tumor tissue and matched adjacent normal kidney tissue and stored at −80°C. All patients were diagnosed with KIRC and did not undergo any antitumor therapy before surgery. The research was authorized by the Medical Ethics Committee of the Southeast University Zhongda Hospital (ZDKYSB077), and each patient gave informed consent.

Total RNA was isolated from KIRC cells and clinical tissues using Total RNA Kit I (50) (OMEGAbiotec, China). Then cDNA was synthesized using the HiScript II Q RT SuperMix (R223-01) reagent kit (vazyme, Nanjing, China). Quantitative real-time PCR (qRT-PCR) was performed using the SYBR green PCR mix (vazyme, Nanjing, China) according to the manufacturer’s instructions. The 2^−ΔΔCT^ calculation method (21, 22), a relative quantification to calculate the proportion of transcripts in a sample, was applied to determine the relative expression levels of the five m6A-related lncRNAs in the prognostic signature. It described the expression levels of the target genes relative to the reference genes. The detailed calculation method of ΔΔCT was as follows: ΔΔCT= (CT_lncRNA_ -CT_GAPDH_) _sample_- (CT_lncRNA_ -CT_GAPDH_) _control_ (The control group in this study was HK-2 cell or normal kidney tissue). GAPDH was employed as the endogenous control. The final results obtained from the 2^−ΔΔCT^ calculation were the relative expression of the target genes. The primer sequences used in the present study were listed in **Table S1**.

### Statistical Analysis

The statistical analysis was performed in R software (version 4.0.2). The Perl programming language (Version 5.30.2) was used for data processing. Kaplan-Meier survival curve analysis with log-rank test was applied to analyze OS. Univariate, Lasso, and multivariate Cox regression analyses were used to evaluate prognostic significance. ROC curve analysis and its AUC value was used to evaluate the reliability and sensitivity of the prognostic signature. P < 0.05 was regarded as statistically significant.

## Results

### Construction and Evaluation of an m6A-Related lncRNAs Prognostic Signature in Training Set

**Figure 1:**
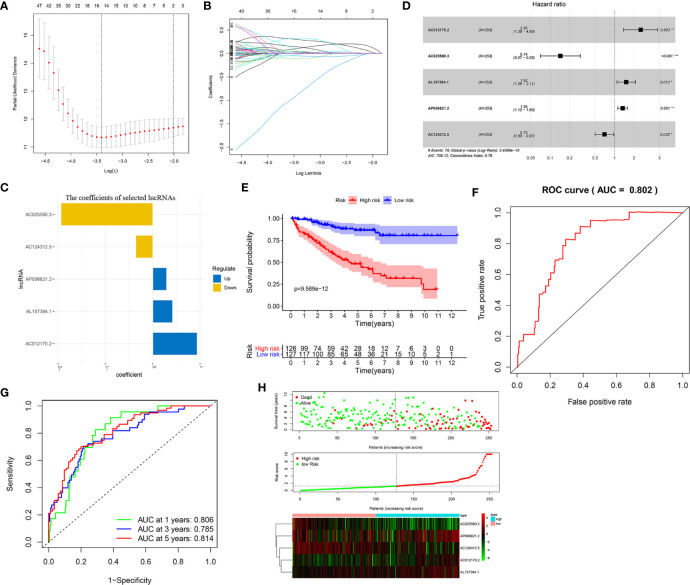
Construction and evaluation of an m6A-related lncRNAs prognostic signature in Training set. **(A–C)** The least absolute shrinkage and selection operator (LASSO) Cox regression analysis was performed to avoid overfitting in training set after univariate Cox regression analysis. Lasso coefficient values and vertical dashed lines were calculated at the best log (lambda) value **(A, B)** and Lasso coefficient profiles of the prognostic-related lncRNAs were displayed **(C)**. **(D)** Forest plot of multivariate cox regression analysis for five prognostic-related lncRNAs. The Hazard Ratio (HR) value and its 95% confidence interval, as well as associated p-value, were showed. These HRs greater than 1 were risk factors, which indicated that high expression of these lncRNAs was unfavorable for prognosis, while HRs less than 1 were protective factors, which indicated that high expression of lncRNAs was favorable for prognosis. **(E)** Kaplan-Meier curves showed that the high-risk group had worse survival probability than the low-risk group in the training set. **(F)** Receiver operating characteristic (ROC) curves for the signature and its AUC value in training set. **(G)** ROC curves and their AUC value represented 1-, 3-, and 5-year predictions in training set. **(H)** Scatter plot showed the correlation between the survival status and risk score of KIRC patients; Risk score distribution plot showed the distribution of high-risk and low-risk KIRC patients; Heatmap of the five m6A-related lncRNA expression profiles showed the expression of risk lncRNAs in high-risk and low-risk group in training set. *p < 0.05; **p < 0.01; ***p < 0.001.

To construct a prognostic prediction signature for KIRC patients, we performed univariate Cox proportional hazards regression analysis of expression of the 753 m6A-related lncRNAs in the training set. Expression of 297 lncRNAs was shown to be significantly associated with the prognosis of KIRC patients. LASSO Cox analysis was applied to eliminate these prognostic-related lncRNAs highly correlated with each other to avoid overfitting, and 15 m6A-related lncRNAs were identified (**Figures 1A, B** **)**. Subsequently, multivariate Cox proportional hazards regression analysis were adopted, and it generated the m6A-related lncRNAs prognostic signature which contained five m6A-related lncRNAs and coefficient of each (**Figure 1C**), using the formula as follows: risk score = 0.935053 * AC012170.2+(−1.93775) * AC025580.3+0.416438 * AL157394.1+0.291862 * AP006621.2+(−0.35955) * AC124312.5. Also, forest plots of multivariate cox regression analysis displayed that AC012170.2, AL157394.1, and AP006621.2 were risk factors for Hazard Radio (HR) >1, whereas AC025580.3 and AC124312.5 were protective factors for HR <1 (**Figure 1D**).

**Figure 2:**
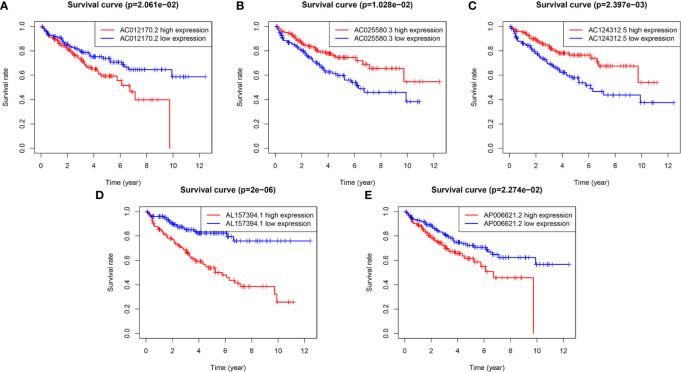
The Kaplan-Meier (K-M) survival curves of five m6A-related lncRNAs in the prognostic signature. **(A, D, E)** The K-M survival curves of AC012170.2, AL157394.1, and AP006621.2 showed high expression group had worse overall survival (OS) than the low expression group in the training set (p < 0.05). **(B, C)** The K-M survival curves of AC025580.3 and AC124312.5 showed high expression group had better OS than the low expression group in the training set (p < 0.05).

To evaluate the reliability and sensitivity of the prognostic risk-related signature, the KIRC patients in the training dataset were assigned to low- and high-risk subgroups based on the median value of risk scores. Kaplan-Meier survival curves were performed and depicted that the survival outcomes of KIRC patients with high-risk subgroup were significantly worse than those with low-risk subgroup in the training set (p < 0.001) (**Figure 1E**). The 3-, 5-year survival rates were 60.7 and 46.2% for the high-risk subgroup and 90.6 and 86.5% for the low-risk subgroup, respectively. ROC curves showed that the AUC value for prognostic risk-related signature was 0.802 (**Figure 1F**). Moreover, the AUC value corresponding to 1, 3, 5 years of survival outcomes were 0.806, 0.785, and 0.814 (**Figure 1G**), which demonstrated that the prognostic risk-related signature harbored a promising ability to predict prognosis in the training set. In addition, scatter plot showed that high-risk score KIRC patients had worse survival times than low-risk score group; the risk Score distribution plot depicted that the high-risk subgroup had higher risk scores than the low-risk subgroup; furthermore, the heatmap showed significant differences in the expression profiles of five prognosis-related lncRNAs between the high-risk and low-risk subgroups (**Figure 1H**). Besides, the Kaplan-Meier survival curves were applied to evaluate prognostic roles of the five prognosis-related lncRNAs, and the results confirmed that higher expression of AC012170.2 (**Figure 2A**), AL157394.1 (**Figure 2D**), and AP006621.2 (**Figure 2E**) and lower expression of AC025580.3 (**Figure 2B**) and AC124312.5 (**Figure 2C**) were linked to poorer survival outcomes (p < 0.05). In summary, the prognostic risk-related signature we constructed had significant reliability and sensitivity in predicting the prognosis of KIRC patients.

### Validation of the m6A-Related lncRNAs Prognostic Signature in Testing Set

**Figure 3:**
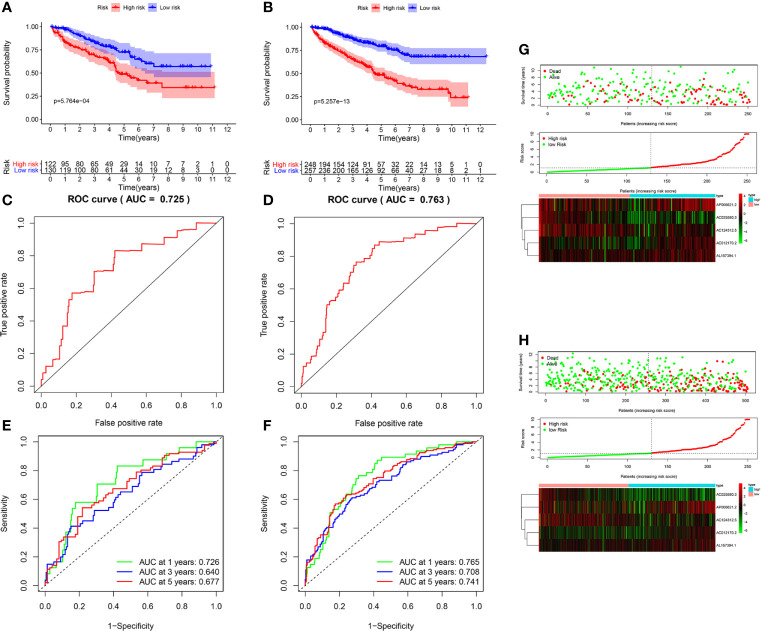
Validation of the prognostic signature for KIRC patients in testing set and overall set. Kaplan-Meier curves showed that the high-risk group had worse overall survival (OS) than the low-risk group in the testing set **(A)** and overall set **(B)**. Receiver operating characteristic (ROC) curves for the prognostic signature and its AUC value in the testing set **(C)** and overall set **(D)**. ROC curves and their AUC value represented 1-, 3-, and 5-year predictions in the testing set **(E)** and overall set **(F)**. Scatter dot plot showed the outcomes between the survival status and risk score of KIRC patients in high- and low-group; Risk score distribution plot showed the distribution of high-risk and low-risk KIRC patients; Heatmap of the five m6A-related lncRNA expression profiles showed the expression of risk lncRNAs in high-risk and low-risk group in the testing set **(G)** and overall set **(H)**, separately.

To further validate the predictive ability of the m6A-related lncRNAs prognostic signature, we calculated the risk scores in both testing set and overall set using the same algorithm for KIRC patients, who were also divided into low- and high-risk subgroups. Kaplan-Meier survival curves displayed that the OS for KIRC patients were consistent with those in the testing set (**Figure 3A**) and overall set (**Figure 3B**) (p < 0.001). The 3-, 5-year survival rates were 67.9 and 46.8% for the high-risk subgroup and 82.1 and 70.7% in the low-risk subgroup in the testing set, and 64.8 and 46.4% for the high-risk subgroup and 86.2 and 78.4% in the low-risk subgroup in the overall set, respectively. ROC curves also indicated that the m6A-related lncRNAs prognostic signature had a reliable predictive capability in the testing set (AUC = 0.725; **Figure 3C**) and overall set (AUC = 0.763; **Figure 3D**). Furthermore, the time-ROC curves and its AUC value also displayed that the prognostic signature had strong prognostic value for KIRC patients in testing set (1-year AUC = 0.726, 3-year AUC = 0.640, 5-year AUC = 0.677; **Figure 3E**) and overall set (1-year AUC = 0.765, 3-year AUC = 0.708, 5-year AUC = 0.741; **Figure 3F**). Besides, the scatter plot and risk score distribution plot also displayed the correlations between survival status and risk score of KIRC patients in high- and low-risk subgroup in the testing set (**Figure 3G**) and overall set (**Figure 3H**). Also, heatmaps showed that the expression profiles of the five prognosis-related lncRNAs were also consistent with those in the training set. These results indicated that the m6A-related lncRNAs prognostic signature had a robust and stable prognostic-predictive ability.

### Clinical Value and Application of the m6A-Related lncRNAs Prognostic Signature

**Figure 4:**
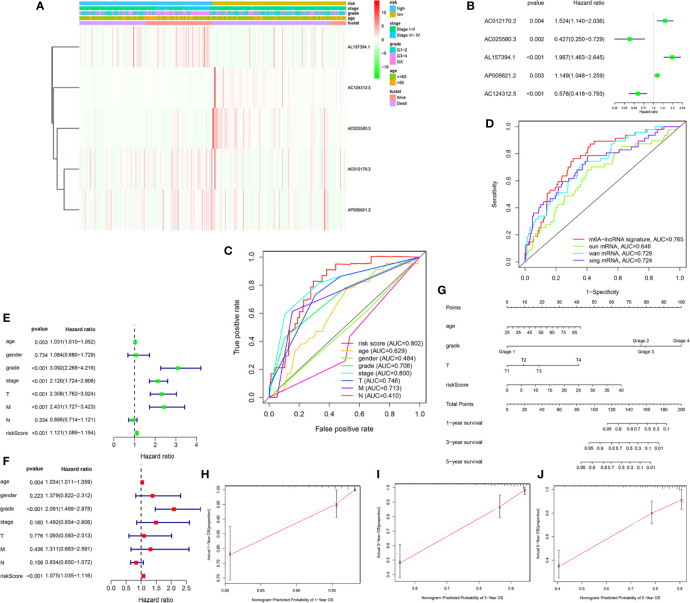
Estimation of clinical Value of the m6A-related lncRNAs prognostic risk signature in KIRC patients. **(A)** The heatmap showed associations between the expression of the five m6A-related lncRNAs in the low- and high-risk group and clinicopathological features, including survival status (alive or dead), age (>60 y or <=60 y), AJCC stages (stages I–II or III–IV), and AJCC grade (1–2, 3–4, or NA) (all p < 0.05) in training set. **(B)** The forest plots showed the prognostic ability of the five m6A-related lncRNAs in the prognostic risk model (p < 0.05). **(C)** The multivariate receiver operating characteristic (ROC) curve showed predictive accuracy of risk score was higher than other clinicopathological features. **(D)** Multivariate ROC curves showed the sensitivity and specificity of the prognostic risk signature were higher than other published biomarkers in predicting the prognosis of KIRC patients. **(E)** The univariate Cox regression analysis showed that risk score and clinicopathological features, included age, grade, AJCC stage, T and M stage were prognostic-related variables. **(F)** The multivariate Cox regression analysis showed risk score, grade, age were independent prognostic factors. **(G)** Construction of a prognostic nomogram based on risk score and prognostic-related clinicopathological parameters to predict 1-, 3-, 5-year overall survival of KIRC patients. **(H–J)** The calibration curves of the nomogram displayed the concordance between predicted and observed 1-, 3-, and 5-year OS.

To access the clinical value and application of the prognostic signature, the risk scores from prognostic signature and clinicopathological characteristics, including age, gender, grade, AJCC stage, TNM stage were integrated. As was shown in **Figure 4A**, the heatmap showed associations between the expression profiles of the five m6A-related lncRNAs and clinicopathological features in the low- and high-risk subgroup. We found that there were significant differences in age, grade, AJCC stage, and survival status between high- and low-risk subgroups (p < 0.05). In addition, forest plots showed the stable prognostic ability of the five m6A-related lncRNAs included in the prognostic risk model (**Figure 4B**). Multivariate ROC curve based on the risk score from prognostic signature and clinicopathologic characteristics indicated that the AUC value for risk score was 0.802, which was higher than the AUC value of age (0.629), gender (0.484), Grade (0.708), AJCC stage (0.800), T stage (0.746), M stage (0.713), N stage (0.410) (**Figure 4C**). Furthermore, we compared the m6A-related lncRNAs prognostic signature (AUC = 0.765) with published prediction models [Sun et al. (2) AUC = 0.646; Wan et al. (23) AUC = 0.729; Xing et al. (24) AUC = 0.724] and found that our signature had higher prediction reliability and sensitivity than other published biomarkers (**Figure 4D**). Subsequently, the univariate (**Figure 4E**) and multivariate Cox regression analysis (**Figure 4F**) were performed and confirmed that risk score, age, grade were independent prognostic factors (p < 0.01). Overall, our results indicated that the prognostic risk score signature could be used independently and reliably to predict survival outcomes in patients with KIRC.

Finally, to develop a quantitative method to predict the prognosis of KIRC patients, we constructed a prognostic nomogram based on risk score and prognostic-related clinicopathological parameters to predict 1-, 3-, 5-year OS of KIRC patients (**Figure 4G**). The C-index value of this nomogram was 0.794. The calibration curve proved that the prognostic nomogram was reliable and accurate (**Figures 4H–J**).

### Stratification Analysis of the m6A-Related lncRNAs Prognostic Signature Based on Prognosis-Related Clinicopathological Features

**Figure 5:**
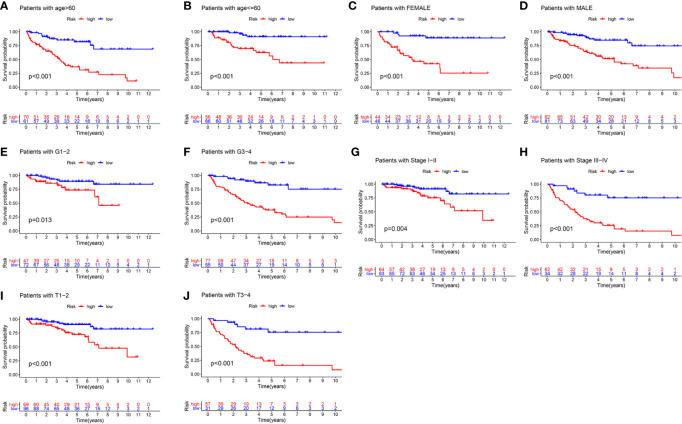
The survival outcomes of the high- and low-risk score subgroup in KIRC patients were stratified by various clinicopathological features. Kaplan-Meier survival curve showed the survival outcomes of high- and low-risk KIRC patients stratified according to age (>60 years *vs.* ≤60 years) **(A, B)**, gender (FEMALE *vs.* MALE) **(C, D)**, AJCC grade (G1–2 *vs.* G3–4) **(E, F)**, stages (stage I–II *vs.* stage III–IV) **(G, H)**, AJCC T stage (T1–2 *vs.* T3–4) **(I, J)**, respectively (all p < 0.05).

To better evaluate the predictive ability of the m6A-related lncRNAs prognostic signature and to validate its ability to predict OS in high-and low-risk subgroups, we performed a stratified analysis based on clinicopathological features, including age (>60 years *vs.* ≤60 years), gender (FEMALE *vs.* MALE), AJCC grade (G1–2 *vs.* G3–4), stages (stage I–II *vs.* stage III–IV), AJCC T stage (T1–2 *vs.* T3–4). Kaplan-Meier survival analyses were performed and results showed that the high-risk subgroup had worse OS compared to the low-risk subgroup in different strata of clinical characteristics (p < 0.05; **Figures 5A–J**).

### GSEA of the High- and Low-Risk Subgroup in KIRC Patients Based on the m6A-Related lncRNAs Prognostic Signature

**Figure 6:**
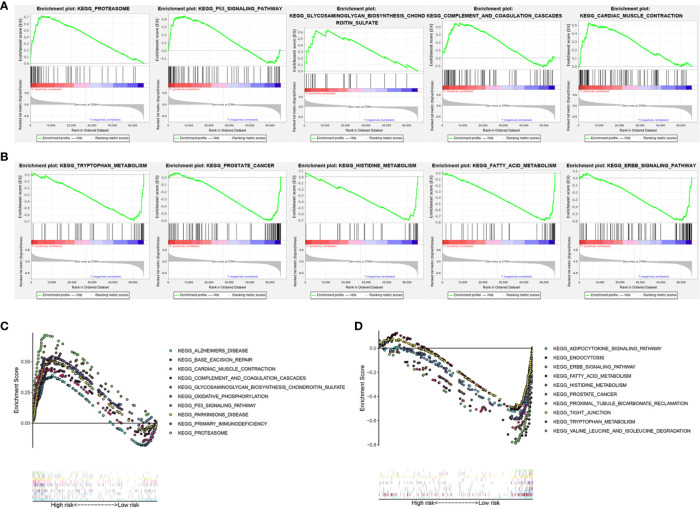
Gene set enrichment analysis (GSEA) of the high- and low-risk subgroup in KIRC patients based on the prognostic signature. **(A)** GSEA showed that the top five tumor hallmarks were enriched in the high-risk group. **(B)** GSEA showed that the top five tumor hallmarks were enriched in the low-risk group. **(C)** The top 10 KEGG signaling pathways in high-risk KIRC patients. **(D)** The top 10 KEGG signaling pathways in low-risk KIRC patients.

To investigate the potential biological processes and pathways involved in molecular heterogeneity, the GSEA was performed between the low- and high-risk subgroups in TCGA cohort. The results displayed that the altered genes in the high-risk subgroups belonged to pathways involving proteasome, cancer-muscle-contraction, glycosaminoglycan-biosynthesis-chondroitin-sulfate, p53-signaling-pathway, complement-and-coagulation-cascades (**Figure 6A**). Besides, the GSEA analysis in the low-risk subgroups related to ERBB-signaling-pathway, tryptophan-metabolism, fatty-acid-metabolism, prostate-cancer, histidine-metabolism (**Figure 6B**). It indicated that activation of pathways in high- or low-risk subgroups could contribute to improving prognosis. As shown in **Figures 6C, D**, the top 10 KEGG signaling pathways in high- or low-risk subgroups were displayed and suggested enrichment scores in the high-risk subgroup were associated with proteasome, while valine-leucine-and-isoleucine-degradation in low-risk subgroup. These findings gave new insights into individualized treatment for different risk subgroups of patients with KIRC.

### Construction of a ceRNA Network and PPI Network Based on WGCNA and Functional Enrichment Analysis

**Figure 7:**
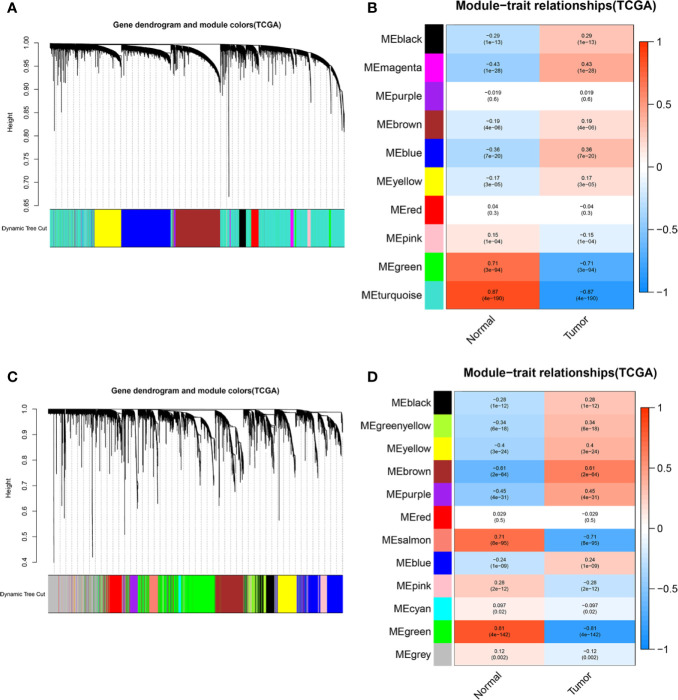
WGCNA was performed to identify modules associated with the clinical traits of KIRC. **(A)** Hierarchical clustering dendrogram of identified lncRNAs in modules of KIRC. **(B)** Heatmaps of the correlation between Eigengene of lncRNAs and clinical traits of KIRC were displayed. Each module with different colors contained the correlation and P-value, and MEturquoise module with the highest correlation coefficient was selected. **(C)** Hierarchical clustering dendrogram of identified mRNAs in modules of KIRC. **(D)** Heatmaps of the correlation between Eigengene of mRNAs and clinical traits of KIRC cancer were displayed. Each module with different colors contained the correlation and P-value, and MEgreen module with the highest correlation coefficient was selected.

**Figure 8:**
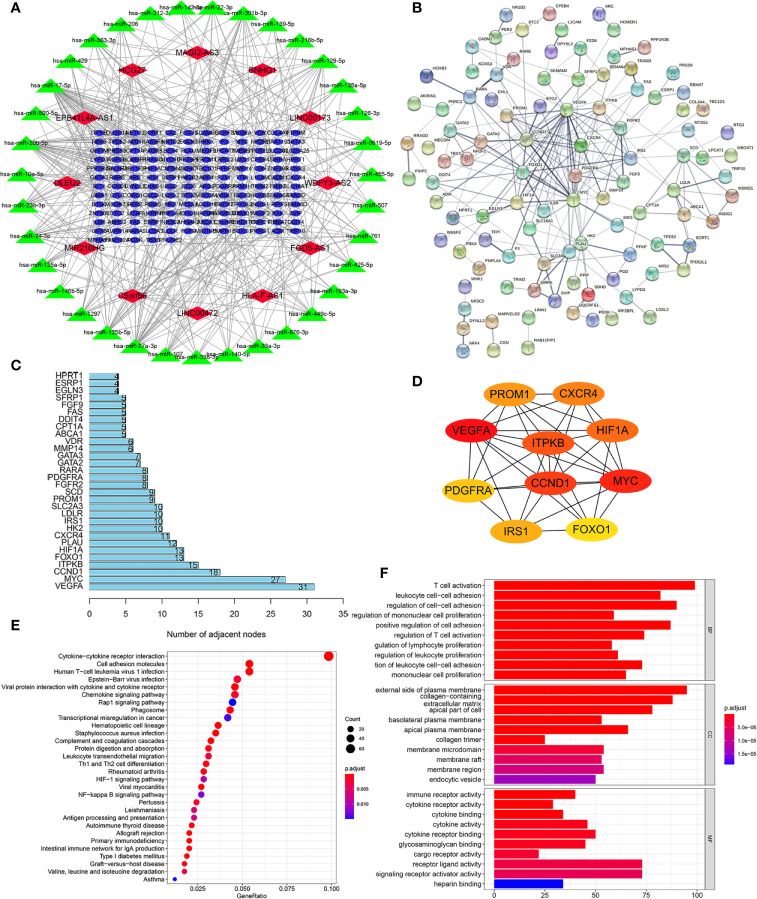
Construction of a lncRNA-miRNA-mRNA ceRNA network and protein-protein interaction (PPI) network, as well as functional enrichment analysis. **(A)** The ceRNA network displayed 12 m6A-related lncRNAs and their sponged miRNAs and targeted mRNAs. **(B)** PPI network of target genes. **(C)** The bar chart showed the number of connecting nodes of target mRNAs in PPI network. **(D)** PPI network of the top 10 hub genes obtained from CytoHubba plugin of Cytoscape. **(E)** Bubble diagram of Kyoto Encyclopedia of Genes and Genomes (KEGG) pathway analysis revealed the enriched signaling pathways of targeted mRNAs. **(F)** Gene Ontology (GO) analysis of targeted mRNAs revealed the enriched biological processes, cell components, and molecular functions.

To elaborate on how m6A-related lncRNAs regulate targeting mRNAs expression by sponging miRNAs in KIRC, we constructed a ceRNA network based on WGCNA and performed PPI analysis using the STRING database. WGCNA was performed to identify lncRNAs in modules associated with the clinical traits of KIRC and MEturquoise module was selected because of the highest correlation coefficient (**Figures 7A, B** **)**. Likewise, these mRNAs in the MEgreen module were selected (**Figures 7C, D** **)**. Then, we constructed a lncRNA-miRNA-mRNA ceRNA network that contained 12 lncRNAs, 35 miRNAs, and 149 mRNAs to investigate the potential biological function of m6A-related lncRNAs (**Figure 8A**). Subsequently, these 149 target mRNAs were applied to implement PPI analysis (**Figure 8B**). The connecting nodes of the top 30 target mRNAs were shown in PPI network, with VEGFA having the most interacting nodes (**Figure 8C**). Besides, we obtained the top 10 hub genes using CytoHubba plugin of Cytoscape software (**Figure 8D**). Ultimately, GO enrichment analysis and KEGG pathway analysis of 149 targeted mRNA were implemented. We found that the top five GO terms for biological processes were T cell activation, leukocyte cell-cell adhesion, regulation of cell-cell adhesion, regulation of mononuclear cell proliferation, positive regulation of cell adhesion; The top five GO terms for cellular components were external side of plasma membrane, collagen-containing extracellular matrix, apical part of cell, basolateral plasma membrane, apical plasma membrane, and the top five GO terms for molecular functions were immune receptor activity, cytokine receptor activity, cytokine binding, cytokine activity, cytokine receptor binding (**Figure 8F**). The top five KEGG signaling pathways were cytokine-cytokine receptor interaction, cell adhesion molecules, human T-cell leukemia virus 1 infection, Epstein-Barr virus infection, viral protein interaction with cytokine and cytokine receptor (**Figure 8E**). These results provided us with new ways to search for potential functions of m6A-related lncRNAs in KIRC.

### Identification of Expression Levels of the Five m6A-Related lncRNAs in KIRC Cells and Clinical Tissue Samples

**Figure 9:**
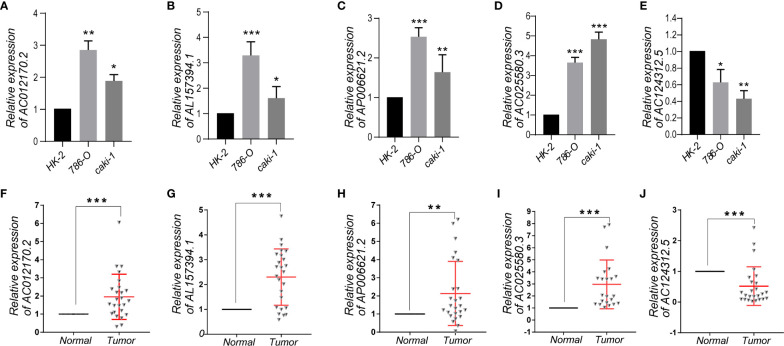
**(A–E)** The expression levels of five m6A-related lncRNAs in the prognostic signature in normal kidney cell and KIRC cells. **(F–J)** The expression levels of five m6A-related lncRNAs in 25 paired KIRC and matched adjacent normal tissues were examined by qRT-PCR. *p < 0.05; **p < 0.01; ***p < 0.001.

To further demonstrate the feasibility of the prognostic signature, we performed qRT-PCR assays in KIRC cells and clinical tissue samples to validate the expression levels of the five m6A-related lncRNAs. We first validated the expression level of the five lncRNAs in normal kidney cells (HK-2 cell) and two KIRC cell lines (786-O, caki-1). The results indicated that the expression level of AC012170.2, AL157394, AP006621.2, and AC025580.3 were significantly increased in KIRC cells compared with normal kidney cells, whereas AC124312.5 was downregulated in KIRC cell (**Figures 9A–E**). The same results were detected in tumor tissue and matched adjacent normal kidney tissue (**Figures 9F–J**). Collectively, these findings further validated the stability and reliability of the m6A-related lncRNAs prognostic signature.

## Discussions

In our present study, we identified five prognostic-associated m6A-related lncRNAs (AC012170.2, AL157394.1, AP006621.2, AC025580.3, and AC124312.5) and constructed an m6A-related lncRNAs prognostic signature that could accurately predict the prognostic outcome of KIRC patients based on TCGA data. Firstly, the KIRC samples have been randomly divided into training set and testing set. Then, univariate Cox proportional hazards regression analysis was applied in the training set, and 297 lncRNAs were found to be associated with the prognosis significantly. Subsequently, LASSO Cox analysis and multivariate Cox proportional hazards regression analysis were adopted. Five prognostic-associated m6A-related lncRNAs were identified as independent prognostic factors for KIRC patients used to construct the prognostic risk score model subsequently. To evaluate the predictive ability of the prognostic signature, we classified the KIRC patients into low- and high-risk subgroups based on the median value of risk scores. Subsequently, we performed Kaplan-Meier survival analysis and confirmed that the high-risk subgroup had a worse OS than low-risk subgroup in the training set, testing set, and overall set. It was consistent with the results of the ROC curves. Moreover, a prognostic nomogram was constructed to predict the OS of KIRC patients quantitatively. Finally, a lncRNA/miRNA/mRNA ceRNA network and a PPI network based on WGCNA were built further to explore the possible biological mechanisms of m6A-related lncRNAs. Besides, GO and KEGG enrichment analysis was performed to validate the main biological functions and downstream pathways of those m6A-related lncRNAs. Collectively, our results indicated that m6A-related lncRNAs prognostic signature had a robust and stable prognostic-predictive ability.

Several studies (2, 25) have reported that m6A-related gene models could predict the prognosis of KIRC patients well, but whether m6A-related lncRNAs prognostic signature could predict the prognosis of KIRC remained unknown. In the present study, we compared the m6A-related lncRNAs prognostic signature with published prediction models and found that our signature had reliable predictive reliability and sensitivity, superior to other published biomarkers. In addition, we developed a prognostic nomogram to accurately predict the prognosis of KIPC patients, which had a comparable predictive ability with the published literature (26, 27). Therefore, this could be a new and useful predictive tool for KIRC patients.

Besides, to access the clinical value of the prognosis signature, we integrated risk scores and clinicopathological characteristics, and performed univariate and multivariate Cox regression analysis and stratification analysis. We found that risk score, age, grade were independent prognostic factors, which indicated that the m6A-lncRNAs prognostic signature could be used independently and reliably to predict OS in KIRC patients. Moreover, stratification analysis demonstrated that the high-risk subgroup had worse OS compared to the low-risk subgroup in different clinical characteristics. It also proved the reliability and usefulness of the prognostic signature.

Then, combined with the expression levels, we analyzed the five m6A-related lncRNAs in the prognostic signature. We found that AC012170.2, AL157394, AP006621.2 were upregulated in tumor tissues compared with normal tissues. Furthermore, AC012170.2, AL157394.1, and AP006621.2 were risk factors, which were upregulated in high-risk subgroup. The Kaplan-Meier survival curves showed that higher expression of AC012170.2, AL157394.1, and AP006621.2 were linked to poorer survival outcomes. These suggested that they might act as tumor suppressors in KIRC. On the contrary, AC124312.5 was downregulated in tumor tissues. Moreover, AC124312.5 were protective factors, which were upregulated in low-risk subgroup. And the lower expression of AC124312.5 was linked to poorer survival outcomes. These suggested that it might act as tumor promoters in KIRC. It gave us insight into their potential role in tumorigenesis and development for KIRC. Also, Xia et al. (28) reported the prognostic role of AP006621.2 and AC025580.3 in KIRC. However, the roles of the remaining three m6A-related lncRNAs in tumors have not been reported. Therefore, our next step will be to further verify its function and mechanism from *in vivo* and *in vitro* experiments.

Our study still had some limitations. Firstly, the dataset we used to construct and validate the m6A-related lncRNAs prognostic signature was obtained from TCGA. We failed to locate suitable external data from other public databases to evaluate the reliability of the model. Second, we only performed preliminary expression studies on these five m6A-related lncRNAs in the signature. However, further functional analysis and mechanistic studies were not carried out. Finally, we were not able to verify its specific biological functions and found the exact signaling pathways.

In conclusion, in the present study, we extracted data from public databases and analyzed the role of m6A-related lncRNAs in KIRC. We successfully constructed a prognostic risk signature based on five m6A-related lncRNAs and validated the reliability and sensitivity of the model. We also established a prognostic nomogram that could quantitatively predict the prognostic outcome of KIRC patients. Besides, the ceRNA network and PPI network were constructed and GO and KEGG functional enrichment analysis was performed, which provided us with new ways to search for potential functions of m6A-related lncRNAs in KIRC.

## Data Availability Statement

The original contributions presented in the study are included in the article/**Supplementary Material**. Further inquiries can be directed to the corresponding author.

## Author Contributions

JY and SS were responsible for study design, data acquisition and analysis, and manuscript writing. JY and WM performed bioinformatics and statistical analyses. QH, CW, ZX, SC, and RL were responsible for collecting clinical samples. JY and WM prepared the figures and tables for the manuscript. BX and MC were responsible for the integrity of the entire study and manuscript review. All authors contributed to the article and approved the submitted version.

## Funding

This study was supported by the National Natural Science Foundation of China (Nos. 81872089, 81370849, 81672551, and 81202034) and Natural Science Foundation of Jiangsu Province (BE2019751, BK20161434, and BK2012336).

## Conflict of Interest

The authors declare that the research was conducted in the absence of any commercial or financial relationships that could be construed as a potential conflict of interest.

## Supplementary Material

Supplementary Figure 1 The flow chart of the study.

Supplementary Figure 2 The heatmap was performed to visualize the differential expression of 35 N6-methyladenosine (m6A) related genes between 489 tumor tissues and 51 normal tissues in The Cancer Genome Atlas (TCGA) prostate cancer cohort. *p < 0.05; **p < 0.01; ***p < 0.001.

## Abbreviations

KIRC, kidney renal clear cell carcinoma; TCGA, The Cancer Genome Atlas; lncRNA, long non-coding RNA; m6A: N6-methyladenosine; ROC, receiver operating characteristic; GO, Gene Ontology; KEGG, Kyoto Encyclopedia of Genes and Genomes; AUC, area under the curve; CI, Confidence intervals; OS, Overall survival; C-index, concordance index; HR, Hazard ratios; AJCC, American Joint Committee on Cancer.
