# Supplementary figures and images for: Identification of an m6A-Related lncRNA Signature for Predicting the Prognosis in Patients With Kidney Renal Clear Cell Carcinoma
Source: Front Oncol. 2021 May 26;11:663263. doi: 10.3389/fonc.2021.663263 (PMC8187870; doi:10.3389/fonc.2021.663263)

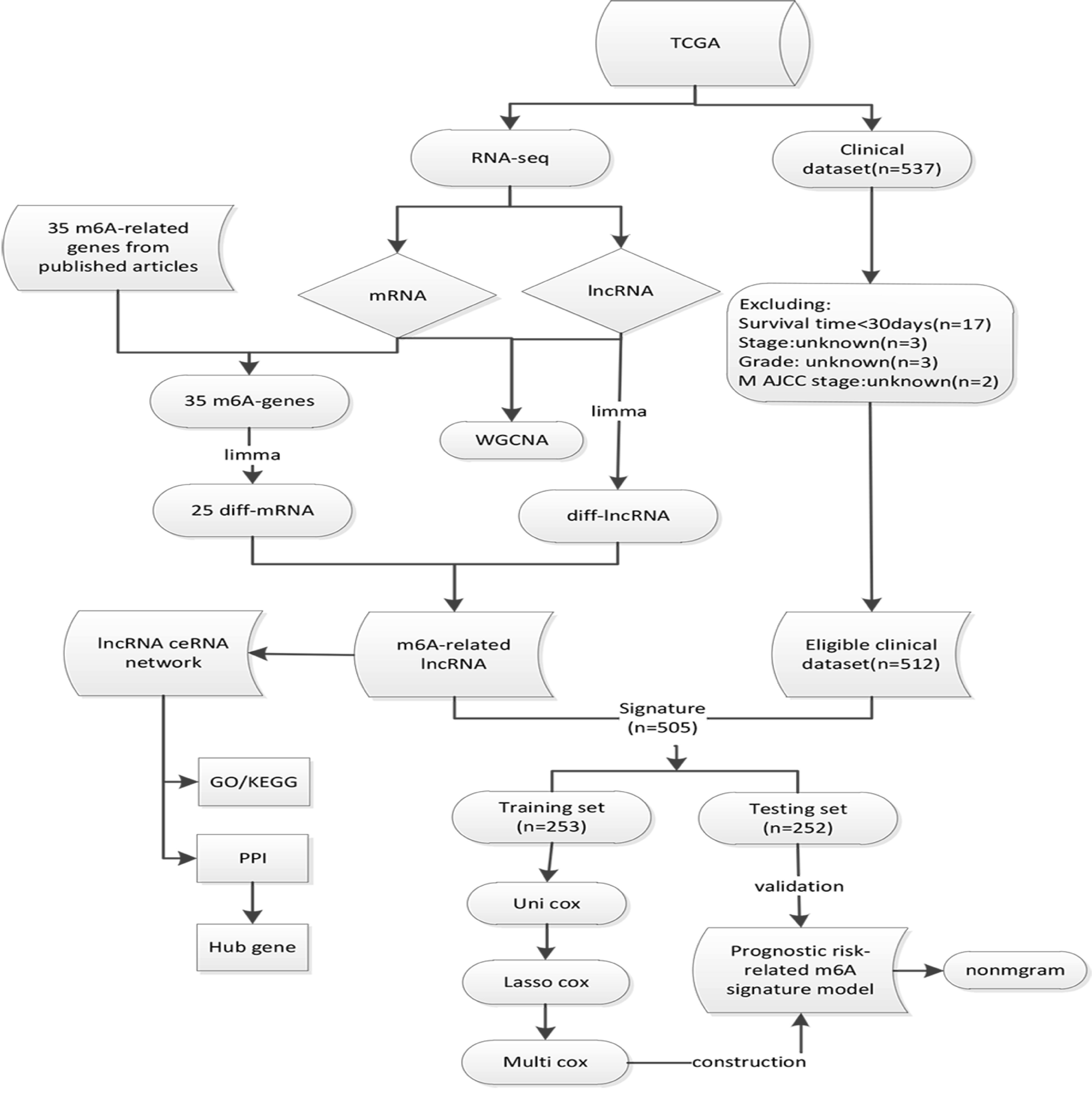

Supplement: Supplementary Figure 1 — The flow chart of the study. [file Image_1.tif]

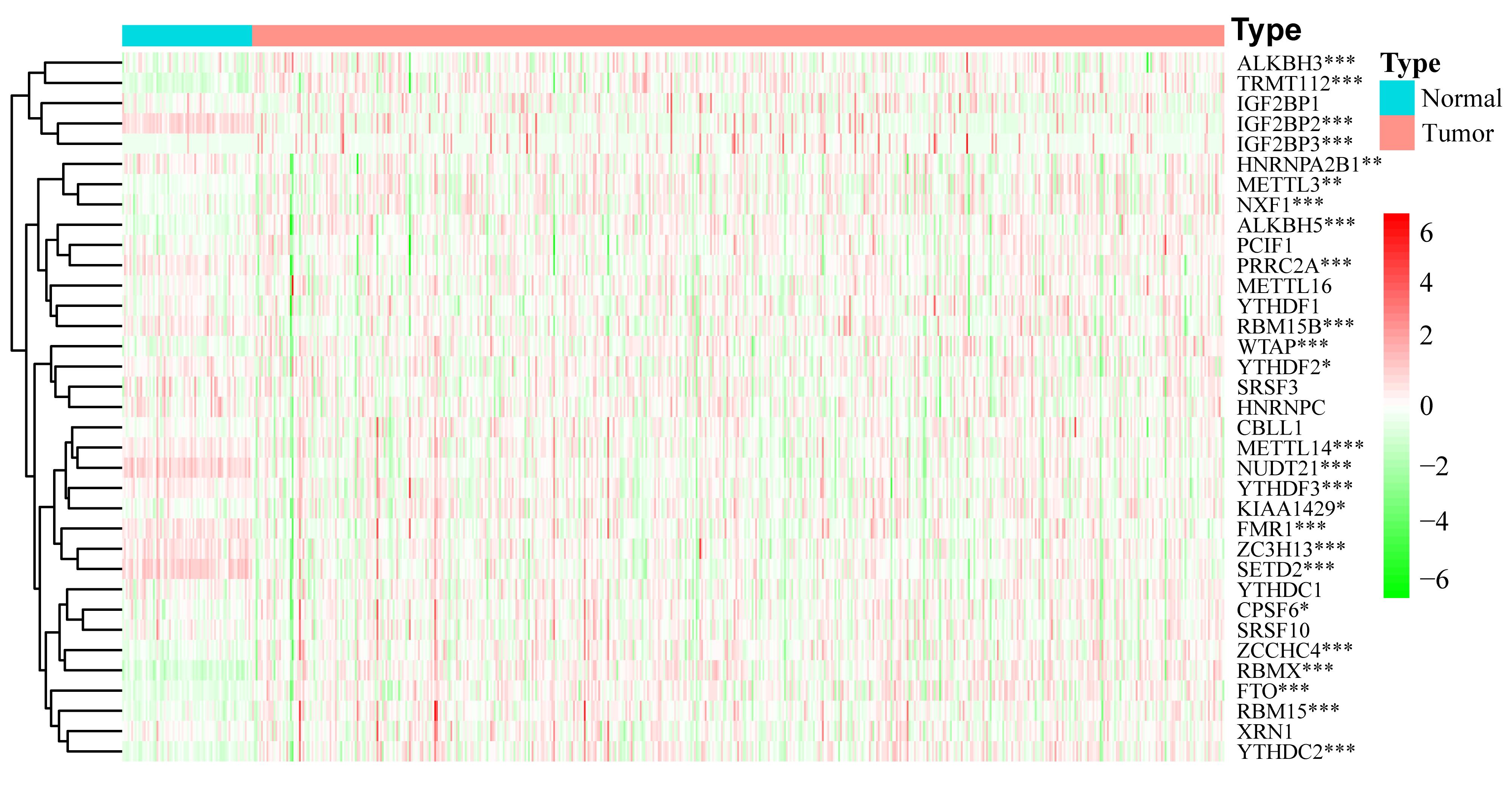

Supplement: Supplementary Figure 2 — The heatmap was performed to visualize the differential expression of 35 N6-methyladenosine (m6A) related genes between 489 tumor tissues and 51 normal tissues in The Cancer Genome Atlas (TCGA) prostate cancer cohort. *p < 0.05; **p < 0.01; ***p < 0.001. [file Image_2.tif]
