# Supplementary material for: Identification of an m6A-Related lncRNA Signature for Predicting the Prognosis in Patients With Kidney Renal Clear Cell Carcinoma
Source: Front Oncol. 2021 May 26;11:663263. doi: 10.3389/fonc.2021.663263 (PMC8187870; doi:10.3389/fonc.2021.663263)
Supplement: Supplementary file 3 [file Table_1.docx]

**Table S1:** The primer sequences used in the present study.

| lncRNA | Forward primer (5′-3′) | Reverse primer (5′-3′) |
| --- | --- | --- |
| AC012170.2 | AACAGCAAACACAAGGTGC | GCTTGAGTGTGATGTTGGG |
| AL157394.1 | CACCTAGTGGCCCCAAGCAT | CTACCCAGTATGCCCACCACA |
| AP006621.2 | AACATCCCCCACAGATGGCT | TCTGCTTCCTGCTTCTGGGT |
| AC025580.3 | ACTGACGGCTTCTGTTGCAC | TGCATAGACCATTGTCACTTCGT |
| AC124312.5 | TCCTTCACGCCATCACAGA | TTGTTGGTGCTCTGTC AACC |
| GAPDH | ACCATCTTCCAGGAGCGAGAT | GGGCAGAGATGATGACCCTTT |
